# Comparative *In Vitro* Activities of SMT19969, a New Antimicrobial Agent, against 162 Strains from 35 Less Frequently Recovered Intestinal Clostridium Species: Implications for Clostridium difficile Recurrence

**DOI:** 10.1128/AAC.02184-13

**Published:** 2014-02

**Authors:** Ellie J. C. Goldstein, Diane M. Citron, Kerin L. Tyrrell

**Affiliations:** a R. M. Alden Research Laboratory, Culver City, California, USA; b David Geffen School of Medicine at UCLA, Los Angeles, California, USA

## Abstract

We determined the comparative activity of SMT19969 (SMT) against 162 strains representing 35 well-characterized Clostridium species in clusters I to XIX and 13 Clostridium species that had no 16S rRNA match. SMT MICs ranged from 0.06 to >512 μg/ml and were not species related. SMT might have less impact on normal gut microbiota than other Clostridium difficile infection (CDI) antimicrobials.

## TEXT

Clostridium difficile infections (CDI) have increased in frequency and severity over the past decade and are a leading cause of hospital-acquired infections, contributing to increased hospital length of stay and costs, as well as associated increased mortality, especially among the elderly (1, 2). Standard therapy has been associated with 20 to 30% relapse rates (3, 4). Consequently, new CDI therapeutic approaches have emerged.

Recurrences of CDI are associated with disruption in the patient microbiome, with changes in richness, evenness, and diversity (5). This antibiotic-induced depletion of normal microbiota allows C. difficile to proliferate, produce toxin, and cause disease. Several investigators have suggested that a reduced impact by antimicrobials on normal flora might lower the risk of recurrent disease, especially on the Bacteroides fragilis group species and Clostridium species cluster XIVa and, to a lesser extent, cluster IV, which contain a large number of butyrate-producing anaerobes (6–8).

SMT19969 (SMT) is a novel, narrow-spectrum, nonabsorbable agent with previously shown activity against C. difficile but with poor activity against B. fragilis (9). Information about its effect on other gut organisms is limited, including data about its activity against the other Clostridium species/clusters. Consequently, we studied the comparative *in vitro* activity of SMT19969 against 162 strains of Clostridium representing 35 well-characterized species and 13 strains with no PCR species match within 8 different Clostridium clusters, especially those of cluster XIVa.

Isolates were recovered from clinical specimens from 1985 to 2013. They were identified by standard methods (10, 11) and by 16S RNA gene sequencing as previously published (12) and stored in 20% skim milk at −70°C. They were taken from the freezer and transferred at least twice on supplemented brucella agar to ensure purity and good growth. Inocula were prepared by direct suspensions of cells into brucella broth to achieve the turbidity of the 0.5 McFarland standard. The final inoculum was ∼10^5^ CFU/spot. Susceptibility to SMT19969, fidaxomicin, vancomycin, and metronidazole was determined using the agar dilution method according to the CLSI approved standard for anaerobes (M11-A8) (13).

The results of this study are shown in Table 1. SMT MICs were variable (range, 0.06 to >512 μg/ml). Resistance (MIC > 32 μg/ml) was not cluster or species related and occurred in Clostridium ramosum (10 of 10 samples), Clostridium cadaveris (2 of 6), Clostridium colicanis (1 of 2), Clostridium glycolicum (2 of 5), Clostridium paraputrificum (6 of 8), Clostridium perfringens (9 of 11), Clostridium rectum (3 of 3), Clostridium sardiniense (1 of 1), Clostridium scindens (1 of 5), Clostridium sordellii (1 of 6), Clostridium sporogenes (3 of 5), and 8 of 13 Clostridium species with no species match by 16S RNA gene sequencing. MICs of ≥32 μg/ml also occurred with fidaxomicin (MIC range of ≤0.03 to >128 μg/ml), but these species were different from the SMT-resistant species. The MIC range for vancomycin was 0.5 to >32 μg/ml and for metronidazole was ≤0.06 to 16 μg/ml, with one or more strains of the unidentifiable Clostridium species showing decreased susceptibility or resistance.

**TABLE 1:** Comparative *in vitro* activity of 162 Clostridium strains analyzed by cluster to SMT19969, fidaxomicin, metronidazole, and vancomycin

RMA no.^*a*^	Species	Clostridial cluster	MIC (μg/ml)			
			SMT19969^*b*^	Fidaxomicin	Metronidazole	Vancomycin
18328	Clostridium baratii	I	>512	≤0.03	1	2
6392	C. baratii-like	I	0.5	≤0.03	1	2
19025	Clostridium butyricum	I	0.25	0.06	0.5	0.5
19848	C. butyricum	I	0.25	≤0.03	0.5	0.5
21418	C. butyricum	I	0.25	0.06	1	0.5
22044	C. butyricum	I	0.25	0.06	1	0.5
22081	C. butyricum	I	0.5	0.125	1	0.5
14198	Clostridium cadaveris	I	1	≤0.03	0.125	2
16516	C. cadaveris	I	2	≤0.03	0.125	2
16863	C. cadaveris	I	1	0.06	0.125	4
18944	C. cadaveris	I	32	≤0.03	0.125	2
19962	C. cadaveris	I	256	0.06	0.125	2
20805	C. cadaveris	I	0.25	≤0.03	0.06	2
6433	Clostridium colicanis	I	0.5	≤0.03	2	>32
6786	C. colicanis	I	64	≤0.03	2	2
15999	Clostridium disporicum	I	0.06	≤0.03	1	0.5
21544	C. disporicum	I	0.25	≤0.03	0.25	0.25
12757	Clostridium fallax	I	0.125	≤0.03	0.5	0.5
21095	C. fallax	I	0.06	≤0.03	1	1
12522	Clostridium novyi A	I	0.25	≤0.03	1	0.5
15199	Clostridium paraputrificum	I	1	0.06	2	1
16518	C. paraputrificum	I	64	≤0.03	2	2
18947	C. paraputrificum	I	64	≤0.03	1	1
21627	C. paraputrificum	I	64	≤0.03	0.5	2
21630	C. paraputrificum	I	64	≤0.03	2	1
22852	C. paraputrificum	I	0.5	≤0.03	2	1
16521B	C. paraputrificum	I	64	≤0.03	1	1
16597A	C. paraputrificum	I	64	≤0.03	2	1
21966	Clostridium perfringens	I	256	≤0.03	1	1
22113	C. perfringens	I	>512	≤0.03	2	1
22244	C. perfringens	I	>512	≤0.03	0.5	1
22245	C. perfringens	I	>512	≤0.03	1	1
22509	C. perfringens	I	>512	0.06	4	1
22671	C. perfringens	I	>512	0.06	4	1
22722	C. perfringens	I	>512	≤0.03	2	1
22810	C. perfringens	I	64	≤0.03	1	1
22842	C. perfringens	I	256	≤0.03	2	1
22885	C. perfringens	I	1	≤0.03	0.5	1
23087	C. perfringens	I	8	≤0.03	4	1
21091	Clostridium sardiniense	I	>512	≤0.03	4	32
9638	Clostridium sporogenes	I	0.5	0.06	0.25	4
10379	C. sporogenes	I	64	0.06	0.25	2
10900	C. sporogenes	I	64	0.06	0.25	4
15061	C. sporogenes	I	64	0.06	0.25	2
16077	C. sporogenes	I	4	≤0.03	≤0.06	2
15329	Clostridium subterminale group	I	0.125	≤0.03	0.25	1
18693	C. subterminale group	I	2	≤0.03	0.5	1
19908	C. subterminale group	I	0.125	≤0.03	0.5	0.5
20775	C. subterminale group	I	0.5	≤0.03	0.25	1
8622B	C. subterminale group	I	≤0.03	≤0.03	0.5	1
14609	Clostridium tertium	I	0.5	≤0.03	1	2
16273	C. tertium	I	1	≤0.03	1	2
18836	C. tertium	I	4	≤0.03	2	2
19847	C. tertium	I	4	≤0.03	1	2
22841	C. tertium	I	0.5	0.06	2	2
18623	Clostridium bartlettii	XI	1	≤0.03	1	2
5262	Clostridium bifermentans	XI	0.125	≤0.03	0.5	0.5
5324	C. bifermentans	XI	0.5	≤0.03	0.5	0.5
9640	C. bifermentans	XI	0.5	≤0.03	0.5	0.5
9897	C. bifermentans	XI	0.5	≤0.03	0.5	0.5
9948	C. bifermentans	XI	0.5	≤0.03	0.5	1
21658	C. bifermentans	XI	0.25	≤0.03	1	0.5
9388B	C. bifermentans	XI	0.25	≤0.03	2	0.5
8910	Clostridium glycolicum	XI	32	≤0.03	0.125	0.5
14467	C. glycolicum	XI	0.5	0.5	0.25	0.5
15023	C. glycolicum	XI	0.5	0.5	0.125	0.5
16312	C. glycolicum	XI	0.5	0.25	0.25	2
7121	C. glycolicum-like	XI	32	1	0.25	0.5
22811	Clostridium mayombei-like	XI	0.5	0.5	1	0.25
16782	Clostridium sordellii	XI	1	≤0.03	2	1
18788	C. sordellii	XI	64	≤0.03	4	1
21861	C. sordellii	XI	16	≤0.03	4	0.5
21976	C. sordellii	XI	1	≤0.03	8	1
22672	C. sordellii	XI	8	0.125	4	1
4634	C. sordellii-like	XI	2	≤0.03	1	1
16057	Clostridium aldenense	XIVa	0.5	64	≤0.06	1
18348	C. aldenense	XIVa	0.5	64	≤0.06	1
18939	C. aldenense	XIVa	0.5	64	≤0.06	1
23550	C. aldenense	XIVa	0.125	64	≤0.06	2
20918A	Clostridium aminovalericum	XIVa	0.25	2	0.25	8
10036	Clostridium bolteae	XIVa	0.25	128	≤0.06	1
18941	C. bolteae	XIVa	0.5	128	0.125	2
21972	C. bolteae	XIVa	0.125	64	0.125	1
22131	C. bolteae	XIVa	0.5	64	≤0.06	1
12934	Clostridium celerecrescens	XIVa	0.125	8	0.5	1
19024	C. celerecrescens	XIVa	0.25	32	0.5	1
19963	C. celerecrescens	XIVa	0.5	32	0.5	1
15980	Clostridium citroniae	XIVa	0.125	64	0.25	1
21971	C. citroniae	XIVa	0.125	128	0.125	1
23088	C. citroniae	XIVa	0.125	64	0.125	1
16102A	C. citroniae	XIVa	0.06	64	≤0.06	1
16521A	C. citroniae	XIVa	0.25	64	≤0.06	1
20713	Clostridium clostridioforme	XIVa	0.125	64	0.25	1
21282	C. clostridioforme	XIVa	0.25	128	≤0.06	1
21626	C. clostridioforme	XIVa	0.25	>128	≤0.06	2
22060	C. clostridioforme	XIVa	0.125	128	≤0.06	2
22084	C. clostridioforme	XIVa	0.25	128	≤0.06	2
18723	Clostridium hathewayi	XIVa	0.125	32	0.25	0.5
20145	C. hathewayi	XIVa	0.125	16	0.125	0.5
20647	C. hathewayi	XIVa	0.5	16	0.5	0.5
21975	C. hathewayi	XIVa	0.125	2	0.125	0.5
2489	Clostridium hylemonae	XIVa	0.06	≤0.03	0.5	1
13503	C. hylemonae	XIVa	0.5	0.25	0.25	2
15944	C. hylemonae	XIVa	0.5	0.25	0.125	2
16423	C. hylemonae	XIVa	0.5	0.25	0.125	2
16895	C. hylemonae	XIVa	0.5	0.25	0.125	2
18591	C. hylemonae	XIVa	0.5	0.25	0.25	2
22200	C. hylemonae	XIVa	0.5	0.25	0.25	2
15073A	C. hylemonae	XIVa	0.5	0.25	0.125	1
10628	Clostridium lavalense	XIVa	0.5	0.06	0.125	1
12736	Clostridium scindens	XIVa	0.125	0.02	0.125	0.5
21863	C. scindens	XIVa	0.06	0.06	0.125	0.5
21878	C. scindens	XIVa	64	1	0.5	0.5
22045	C. scindens	XIVa	0.125	0.06	0.25	0.5
22624	C. scindens	XIVa	0.25	1	0.25	0.5
20753	Clostridium symbiosum	XIVa	0.25	4	0.125	1
21214	C. symbiosum	XIVa	0.5	2	0.125	0.5
21868	C. symbiosum	XIVa	1	8	0.25	1
22082	C. symbiosum	XIVa	0.25	2	0.25	1
22366	C. symbiosum	XIVa	0.125	2	0.125	1
20132	Clostridium xylanolyticum	XIVa	0.125	16	1	0.5
15167	Clostridium lactatifermentans	XIVb	0.06	0.06	0.25	>32
5491	Clostridium innocuum	XVI	0.25	>128	2	8
5615	C. innocuum	XVI	0.25	>128	1	16
20638	C. innocuum	XVI	1	256	0.5	16
20645	C. innocuum	XVI	0.25	256	0.5	16
20648	C. innocuum	XVI	0.25	128	0.5	16
20913	C. innocuum	XVI	0.25	256	1	16
21213	C. innocuum	XVI	0.06	256	1	16
21737	C. innocuum	XVI	1	256	1	16
21860	C. innocuum	XVI	0.25	256	1	16
21903	C. innocuum	XVI	0.25	256	16	16
22441	C. innocuum	XVI	0.25	512	0.5	16
23130	C. innocuum	XVI	0.125	128	2	16
10072	Clostridium rectum-like	XIX	>512	>128	0.5	>32
14707	C. rectum-like	XIX	>512	>128	1	>32
16549	C. rectum-like	XIX	32	>128	0.125	>32
20917	Clostridium ramosum	XVIII	>512	>512	0.5	4
21212	C. ramosum	XVIII	>512	>512	0.5	4
21215	C. ramosum	XVIII	>512	>512	0.5	4
21414	C. ramosum	XVIII	>512	>512	8	4
21738	C. ramosum	XVIII	128	>512	0.5	4
21862	C. ramosum	XVIII	>512	>512	0.5	4
21902	C. ramosum	XVIII	512	>512	0.5	4
21974	C. ramosum	XVIII	512	>512	1	4
22193	C. ramosum	XVIII	>512	>512	1	4
22623	C. ramosum	XVIII	>512	>512	1	4
705	Clostridium species		0.5	≤0.03	0.125	1
9906	Clostridium species		>512	129	0.125	32
10271	Clostridium species		1	≤0.03	2	0.5
14157	Clostridium species		32	>128	2	>32
16187	Clostridium species		>512	>128	2	>32
16338	Clostridium species		>512	>128	2	>32
19909	Clostridium species		0.25	>128	16	16
21472	Clostridium species		32	>128	4	>32
21876	Clostridium species		0.25	≤0.03	1	8
22256	Clostridium species		32	≤0.03	0.25	1
22279	Clostridium species		32	≤0.03	0.25	1
15596B	Clostridium species		0.06	≤0.03	0.05	1
18576W	Clostridium species		32	0.06	0.125	2
16034	Flavonifractor plautii		0.06	≤0.03	0.25	8
22112	Robinsoniella species		0.06	2	2	2

a RMA, R.M. Alden Research Laboratory number.

b SMT19969, Summit 19969.

Louie et al. (7) suggested that poor *in vitro* activity against aerobic and facultative Gram-negative bacteria, Bacteroides species, and other Gram-negative anaerobes would result in a “reduced ecological impact.” They performed fecal quantitative counts of Bacteroides species on patients receiving either vancomycin or fidaxomicin in a phase II trial and noted that fidaxomicin's reduced activity was less suppressive. Tannock et al. (5) extended these observations on the fecal microbiota using temporal temperature gradient electrophoresis (TTGE) and quantification of phylogenetic groups using fluorescent *in situ* hybridization and flow cytometry (FISH/FC). In contrast to vancomycin, clostridial cluster XIVa and IV populations increased during and after fidaxomicin treatment. They postulated that this effect of these clusters and Bifidobacterium spp. might explain the reduced relapse rate of fidaxomicin in clinical trials.

Antharam et al. (6) studied the distal fecal flora of 39 patients with CDI and compared them to those of 36 C. difficile-colonized patients and 40 healthy controls. They found that there was a “paucity of Firmicutes sequences in the aggregate gut microbiota” in the CDI and C. difficile-colonized patients compared to in controls. The majority (68.4%) of Firmicutes were clostridia, and “strikingly members of Clostridium cluster XIVa and to a lesser extent cluster IV” were depleted in those CDI and colonized patients. They suggested that “mechanistic studies focusing on the functional roles of these organisms in diarrheal diseases and C. difficile colonization resistance” be performed.

Previously, Goldstein et al. (9) studied the comparative *in vitro* activity of SMT19969 against 174 Gram-positive and 136 Gram-negative intestinal anaerobes and 40 Gram-positive aerobes. SMT19969 was generally less active against Gram-negative anaerobes, especially the Bacteroides fragilis group species, than vancomycin and metronidazole, suggesting a lesser impact on the normal intestinal microbiota that maintain colonization resistance. SMT19969 showed limited activity against other Gram-positive anaerobes, including Bifidobacterium species, Eggerthella lenta, Finegoldia magna, and Peptostreptococcus anaerobius, with MIC_90_ values of >512, >512, 64, and 64 μg/ml, respectively. This suggested that SMT19969's selective activity makes it an excellent candidate for therapy of CDI.

Our current study extends these observations to 162 Clostridium strains representing 35 species within 8 clusters. Clostridium species showed varied susceptibility to SMT19969. Clostridium innocuum (cluster XVII) was susceptible (MIC_90_ of 1 μg/ml) and C. ramosum (cluster XVI) and C. perfringens (cluster I) were nonsusceptible (MIC_90_ of >512 μg/ml) to SMT19969. Against Clostridium cluster XIVa, the MICs ranged from 0.125 to 64 μg/ml and were species specific. Comparatively, XIVa isolates, except for Clostridium hylemonae, Clostridium lavalense, and some C. scindens isolates, had higher MICs to fidaxomicin (0.006 to >128 μg/ml) and vancomycin (0.5 to 2 μg/ml) and lower MICs to metronidazole (0.05 to 1 μg/ml). SMT19969 had higher MICs than fidaxomicin against C. paraputrificum (cluster I) and C. sordellii (cluster XI).

These data show that SMT199969's activity was variable according to Clostridium species and strains within species. Coupled with its lack of activity against B. fragilis and aerobic enteric flora, it might have a lesser impact than other antimicrobials used for CDI therapy on the normal gut microbiota that maintains colonization resistance. Further evaluation by clinical trials seems warranted.
